# Development and validation of vectors containing multiple siRNA expression cassettes for maximizing the efficiency of gene silencing

**DOI:** 10.1186/1472-6750-6-50

**Published:** 2006-12-22

**Authors:** Shunqing Wang, Zhenqi Shi, Wei Liu, Joel Jules, Xu Feng

**Affiliations:** 1 Department of Pathology, University of Alabama at Birmingham, Birmingham, AL 35294, USA; 2 Department of Hematology, First Municipal People's Hospital, Guangzhou, Guangdong, China

## Abstract

**Background:**

RNA interference (RNAi) was originally identified as a biological process in which short double-stranded RNA (dsRNA) suppress the expression of genes complimentary to the dsRNA. This cellular intrinsic gene silencing mechanism has subsequently been developed as a useful tool for studies of gene function. A major strategy for producing small interfering RNA (siRNA) in cultured cells involves the use of siRNA expression vectors in which a RNA polymerase III (Pol III) promoter and transcription stop signal are designed to constitute a functional siRNA expression cassette for production of siRNA. However, most of the available vectors contain only one siRNA expression cassette.

**Results:**

In order to maximize the efficiency and versatility of the vector-based siRNA approach, we have developed vectors containing multiple (up to six) tandem siRNA expression cassettes. Moreover, we demonstrated that these vectors can be used not only to produce different siRNA to simultaneously suppress the expression of multiple genes but also to maximize the silencing of a singe gene.

**Conclusion:**

The vectors containing multiple siRNA expression cassettes can serve as useful tools for maximizing the efficiency of gene silencing.

## Background

RNAi is a newly identified biological process in which specific sequence of short dsRNA (~22 bp) knock down the expression of genes complimentary to the dsRNA. RNAi was initially discovered in plants, worms and fruit flies as an important biological mechanism controlling gene expression and development [1]. It was later recognized that short dsRNA complementary to particular messenger RNA (mRNA) can specifically decrease the levels of the mRNA through the RNAi pathway in mammalian systems as well [2]. In vivo, siRNA are generated from long precursor molecules by a specific RNase III-like endonuclease called Dicer [3]. The siRNA is subsequently incorporated into the RNA induced silencing complex (RISC), a protein/RNA complex. The siRNA allows specific recognition of the mRNA target and the endonuclease activity of the RISC cleaves and destroys mRNA, resulting in decreased levels of the mRNA [3].

Moreover, RNAi, a cellular intrinsic gene silencing mechanism, has been developed to knock down gene expression in a variety of transformed and primary mammalian cells [4,5]. As such, the newly emerged siRNA technology represents a valuable genetic tool for functional genomics and cell signaling pathway analysis in the post-genome sequence era [6]. Currently, there are two major strategies for getting siRNA into mammalian cells to suppress the expression of target genes. The first strategy involves *in vitro *preparation of siRNA and subsequent delivery of the *in vitro *prepared siRNAs into cells by means of transient transfection [4]. siRNA are often prepared in vitro by chemical synthesis, in vitro transcription or digestion of long dsRNA by RNase III-like endonucleases such as Dicer.

The second strategy involves the use of siRNA expression vectors and is thus regarded as vector-based siRNA approach [7,8]. siRNA expression vectors are primarily plasmids or viral vectors designed for producing siRNA within cultured mammalian cells after the vectors are delivered into the cells. A typical siRNA expression vector contains a functional siRNA expression unit (also referred to as siRNA expression cassette). A siRNA expression cassette requires Pol III promoter such as U6 promoter (the promoter for U6 snRNA which is involved in transcript splicing) and H1 promoter (the promoter for H1 RNA which is a component of RNase P) [9]. In addition, two unique restriction sites are created downstream of the promoter for insertion of a small DNA insert encoding a short hairpin RNA targeting the gene of interest. A transcription stop signal for Pol III (namely, TTTTT) is included at the 3' end of the DNA insert [8]. Once the insert-containing vectors are delivered into mammalian cells, they express short hairpin RNA. The short hairpin RNA are then processed by Dicer into ~22 nt siRNA, which activate the RISC complex to cleave the mRNA target and thus inactivate the gene function [8].

Although the vector-based siRNA approach is widely used, currently most vectors contain only one single siRNA expression cassette. In order to maximize the efficiency and versatility of the vector-based siRNA approach, using an efficient cloning strategy we have developed a series of siRNA expression vectors containing multiple tandem siRNA cassettes (up to six). More importantly, we have also carried out proof-of-concept experiments to demonstrate that the tandem siRNA cassettes are functional and capable of producing different siRNA. As a result, these vectors can be used to produce different siRNA to simultaneously knock down the expression of multiple genes. Moreover, these vectors can also be used to maximize the silencing of a single gene by producing different siRNA targeting the different regions of the gene or producing multiple copies of siRNA targeting a single region of the gene.

## Results

### Development of vectors containing multiple siRNA expression cassettes

**Figure 1:**
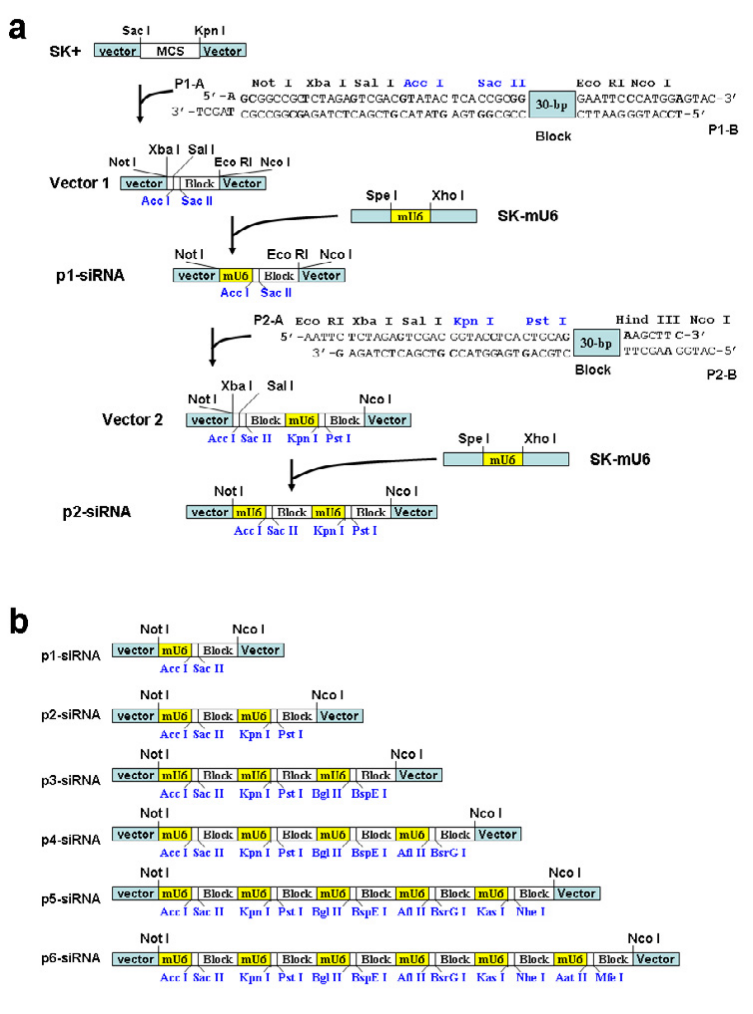
**Cloning strategy for constructing vectors containing multiple siRNA expression cassettes**. a) Detailed procedures for constructing the vectors containing a single siRNA expression cassette (p1-siRNA) or two siRNA cassettes (p2-siRNA). SK+: pBluescript II SK+; MCS: multiple cloning sites; mU6: mouse U6 promoter. The restriction sites in blue highlight the cloning sites for insertion of DNA duplex encoding siRNA. b) Schematic diagram showing the six vectors containing multiple siRNA expression cassettes: p1-siRNA, p2-siRNA, p3-siRNA, p4-siRNA, p5-siRNA and p6-siRNA. Each cassette contains a mouse U6 promoter (mU6), a pair of cloning sites for insertion of DNA duplex encoding siRNA (highlighted in blue) and a block which is included to prevent potential cross-interference among these cassettes. Detailed procedures for constructing p3-siRNA, p4-siRNA, p5-siRNA and p6-siRNA are provided (see Additional file 1).

To develop vectors containing multiple siRNA expression cassettes, we used the cloning vector pBluescript II SK+ (SK+) from STRATAGENE (La Jolla, CA) and followed the cloning strategy detailed in Fig. 1a and additional files (see Additional file 1). SK+ was double digested with Sac I and Kpn I to remove the multiple cloning sites (MCS). A pair of oligonucleotides (P1-A and P1-B) were synthesized and then annealed to form a short DNA duplex (~80-bp). This DNA duplex contains numerous restriction sites required for subsequent cloning steps. Importantly, the DNA duplex starts with a cohesive end (3'-TCGA T-5') which can recombine with the Sac I cohesive end (5'-G AGCT-3). However, once recombined, they give rise to a sequence (5'-GAGCTA-3') which is no longer a Sac I restriction site. Similarly, the duplex DNA ends with a cohesive end which can recombine with the Kpn I cohesive end. But the resulting sequence is no longer a Kpn I site. Consequently, the insertion of the DNA duplex into the cut SK+ gave rise to Vector 1, which contains Not I, Xba I, Sal I, Acc I, Sac I, a block, Eco RI and Nco I (Fig. 1a). A mouse U6 promoter (mU6) was amplified by PCR using mouse genomic DNA and cloned into SK+ between Spe I and Xho I to prepare SK-mU6. mU6 was cut out from SK-mU6 with Spe I and Xho I and cloned into Vector 1 between Xba I and Sal I (Fig. 1a). Notably, although the cohesive ends generated by Xba I and Spe I are compatible, once religated together, they will give rise to a sequence which is no longer recognized by neither Xba I nor Spe I. Similarly, the cohesive ends generated by Sal I and Xho I are compatible, but religation of the ends result in a sequence which is no longer recognized by neither Sal I nor Xho I. This cloning strategy is important since it destroys the Xba I and Sal I once the mU6 is cloned, thus allowing the subsequent insertion of additional mU6 promoters to the vector to generate multiple siRNA expression cassettes. The insertion of mU6 into Vector 1 resulted in a vector containing one siRNA expression cassette, p1-siRNA (Fig. 1a).

To develop a vector containing two siRNA expression cassettes, a pair of oligonucleotides (P2-A and P2-B) was used (Fig. 1a). This short DNA duplex also contains numerous restriction sites including Xba I and Sal I for subsequent addition of mU6 and Kpn I and Pst I for future cloning of DNA insert encoding siRNA. Moreover, the DNA duplex starts with the Eco RI cohesive site and ends with the Nco I cohesive site for insertion into p1-siRNA to generate Vector 2. Then, mU6 was cloned into Vector 2 to produce p2-siRNA. Using a similar cloning strategy, we also prepared four additional vectors: p3-siRNA, p4-siRNA, p5-siRNA and p6-siRNA, which contain 3, 4, 5 and 6 siRNA expression cassettes, respectively (see Additional file 1). Each cassette consists of a mouse U6 promoter, a pair of cloning sites and a block (Fig. 1b). When oligonucleotides designed for producing siRNA are cloned between the cloning sites in each cassette, mouse U6 promoter will drive the production of siRNA. The blocks are used to minimize potential cross-interference among these cassettes.

### The vectors with multiple siRNA expression cassettes can be used to simultaneously inhibit the expression of different genes

**Figure 2:**
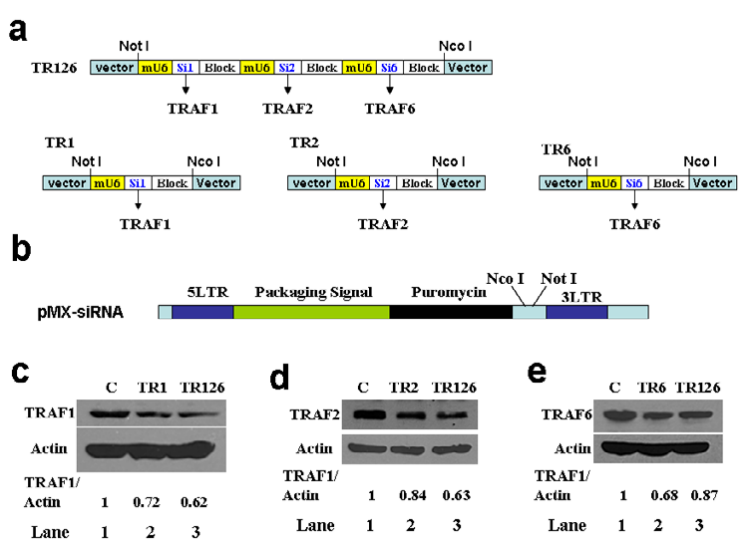
**p3-siRNA is able to produce three different siRNAs to simultaneously suppress the expression of three different genes**. a) Construction of TR126 and three control vectors: TR1, TR2 and TR3. Sequence of Si1, Si2 and Si6 are shown in additional files (Additional file 2). b) Schematic structure of retroviral vector pMX-siRNA. c) – e) Western analysis showing that TR126 is able to suppress the expression of TRAF1 c); TRAF2 d) and TRAF6 e). C: uninfected BMMs controls. Actin was probed as loading control. The expression levels of TRAF proteins were normalized to that of actin. These assays were independently repeated three times and a representative data is shown.

To determine whether these vectors can be used to simultaneously inhibit the expression of multiple genes, we tested the ability of p3-siRNA to produce three different siRNA to target three different genes. In our laboratory, we are primarily working with mouse bone marrow macrophages (BMMs) and BMMs express various tumor necrosis factor-associated factors (TRAF) including TRAF1, 2 and 6 [10]. To this end, we obtained effective siRNA sequences targeting mRNA encoding TRAF1, 2 and 6 by testing various putative sequences identified using the siRNA target finder provided by Ambion (Austin, TX) (data not shown). We synthesized three pairs of oligonucleotides and annealed them to form three DNA duplexes: Si1, Si2 and Si6 which encode siRNA targeting TRAF1, TRAF2 and TRAF6, respectively (see Additional file 2). Si1, Si2 and Si6 were cloned into p3-siRNA to construct TR126 (Fig. 2a). In addition, we also prepared control constructs TR1, TR2 and TR6, each containing a single siRNA expression cassette that express siRNA targeting TRAF1, TRAF2 or TRAF6, respectively (Fig. 2a).

Since BMMs are not transfectable, we have been using retrovirus technology to transduce genes into primary BMMs in our laboratory [11,12]. Thus, we subcloned TR1, TR2, TR6 or TR126 into a retroviral vector called pMX-siRNA vector between Not I and Nco I (Fig. 2b). The virus supernatants prepared using the retroviral vectors were used to infect BMMs and the positively infected cells were selected with puromycin. The expression levels of the three TRAF proteins in BMMs infected with the virus encoding TR1, TR2, TR6 or TR126 were determined by Western analysis. The data indicated that TR1, TR2 and TR6 significantly inhibited the expression of TRAFs 1, 2 and 6, respectively (lane 1 in Fig. 2c,2d and 2e). Importantly, TR126 was also able to significantly suppress the expression of TRAF1, TRAF2 and TRAF6 (lane 2 in Fig. 2c,2d and 2e), indicating that the three siRNA cassettes in p3-siRNA vector are all functional and that p3-siRNA vector is able to simultaneously produce 3 different siRNA to knock down the expression of three different genes. The assays shown in Fig. 2 were performed with p3-siRNA and served as proof-of-concept experiments. Based on these assays, we think that other vectors such as p2-siRNA, p4-siRNA, p5-siRNA and p6-siRNA shown in Fig. 1b should also be able to produce different siRNA to inhibit the expression of different number of genes.

### The vectors with multiple siRNA expression cassettes can also be used to maximize the efficiency of gene silencing

**Figure 3:**
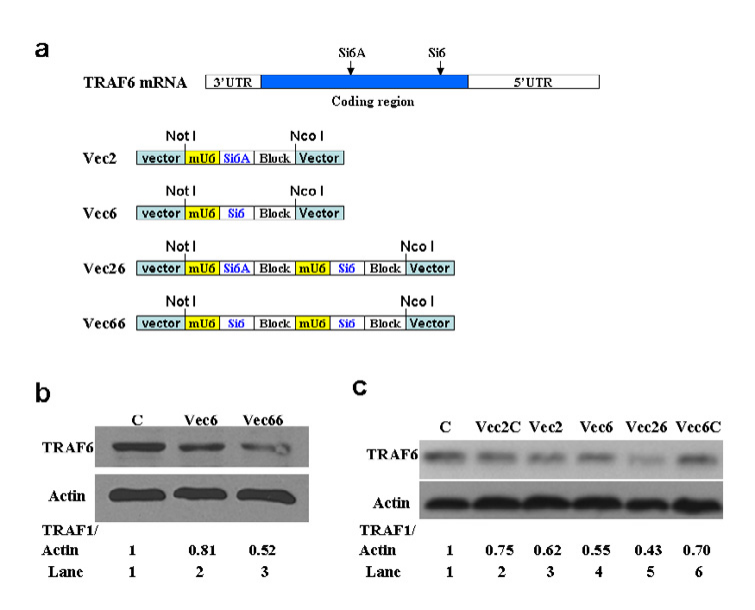
**p2-siRNA is able to produce two different siRNAs targeting different regions of TRAF6 mRNA or two copies of the same siRNA to increase the efficiency of gene silencing**. a) Construction and schematic diagram of Vec2, Vec6, Vec26 and Vec66. In addition, schematic location of sequence Si6A and Si6 are also depicted. UTR: untranslated region. Sequence of Si6 and Si6A are shown in additional files (see Additional file 2). b) Western analysis showing that Vec66 exerts higher inhibitory effect on TRAF6 expression than Vec6. C: uninfected BMMs control. Actin was probed as loading control. The expression levels of TRAF proteins were normalized to that of actin. These Western blot assays were independently repeated three times and a representative data is shown. c) Western analysis showing that Vec26 exerts higher inhibitory effect on TRAF6 expression than Vec2 or Vec6. C: uninfected BMMs control. Vec2C: control containing the scrambled Si6A sequence. Vec6C: control containing the scrambled Si6 sequence.

We next determined whether these vectors can be used to maximize the efficiency of gene silencing by producing siRNA targeting different regions of a gene or by producing more copies of a single siRNA targeting the same region of a gene. To this end, we used Si6 which was able to suppress TRAF6 expression (Fig. 2e). In addition, we identified another effective siRNA sequence (Si6A) targeting a different region of TRAF6 mRNA (Fig. 3a) (data not shown). We used these siRNA sequences to prepare 4 vectors: Vec2, Vec6, Vec26 and Vec66. Vec2 and Vec6 were constructed by cloning a DNA insert encoding Si6A or Si6 into p1-siRNA. Vec2 and Vec6 were used as controls. Vec26 was prepared by ligating both the DNA insert encoding Si6A and the one encoding Si6 into p2-siRNA. The DNA insert encoding Si6 was cloned into both siRNA expression cassettes in p2-siRNA to give rise to Vec66 (Fig. 3a).

We then subcloned Vec2, Vec6, Vec26 and Vec66 into pMX-siRNA vector (Fig. 2b) and virus supernatants prepared using the resulting retroviral vectors were used to infect BMMs. The expression levels of TRAF6 were determined by Western analysis. As shown in Fig. 3b, the production of Si6 by two siRNA cassettes (Vec66) led to a more profound inhibitory effect on TRAF6 expression than single siRNA cassette (Fig. 3b), indicating that the vectors containing multiple siRNA cassettes may also be able to maximize the efficiency of gene silencing by expressing more copies of siRNA targeting the same region of a gene.

Moreover, the simultaneous production of both Si6A and Si6 by Vec26 exerted higher inhibitory effect on the TRAF6 expression (lane 5, Fig. 3c) than the single siRNA (lanes 3 and 4), supporting that the vectors with multiple siRNA expression cassettes have potential to increase the efficiency of gene silencing by expressing different siRNA targeting distinct regions of a gene. Notably, in the previous assays, we used uninfected BMMs as controls. Given the consideration that retroviral infection may cause non-specific inhibitory effect, we included additional controls (Vec2C and Vec6C) in Fig. 3c. Vec2C contains the scrambled Si6A sequence while Vec6C has the scrambled Si6 sequence. Although these controls showed that retroviral infection indeed caused non-specific inhibitory effect, the data do not affect our conclusion that the vectors with multiple siRNA expression cassettes have potential to increase the efficiency of gene silencing by expressing different siRNA targeting distinct regions of a gene. Importantly, the same notion applies to other assays in our current study.

## Discussion

Since the development of siRNA as a genetic tool for gene function studies, two major strategies have been used for getting sufficient amount of siRNA into cultured mammalian cells to knock down gene expression. The first strategy involves direct delivery of the *in vitro *prepared siRNA into mammalian cells by means of transient transfection [4,5]. The major advantage of this strategy is that a large amount of siRNA may be prepared in vitro and then delivered by transient transfection into cells to induce a high magnitude of gene silencing if the cells can be easily and efficiently transfected. However, this strategy inherits two disadvantages: first, the gene silencing using this strategy is transient. Secondly, since some mammalian cells, especially primary cells, can not be efficiently transfected by the current transfection methods, the use of this strategy is largely limited to transfectable cells.

The second strategy is to use siRNA expression vectors [7-9]. The vector-based siRNA approach has several advantages: first, a selection marker (i.e. an antibiotic resistance gene) may be included in the vector for subsequent selection of positively transfected cells to maximize the effect of the gene silencing. Secondly, stable transfectants may be generated using the vector-based siRNA approach to achieve long-term gene silencing. Alternatively, siRNA expression cassettes can be incorporated into viral vectors (retrovirus or adenovirus) to express siRNA in non-transfectable cells, especially primary cells. However, despite its increasing popularity, the vector-based approach has not been fully developed to harness its ultimate potential as a tool for gene silencing, primarily because most available vectors utilize only one siRNA expression cassette.

**Figure 4:**
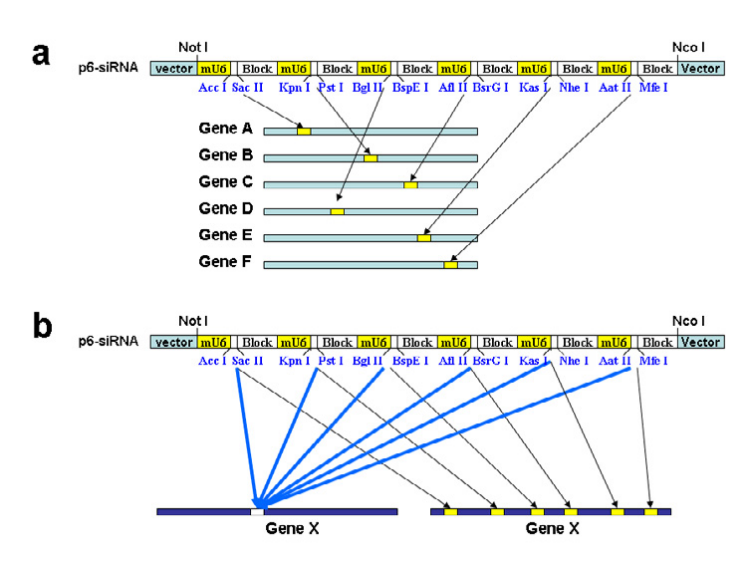
**Diagram depicting the ability of vectors containing multiple siRNA expression cassettes to simultaneously suppress the expression of multiple genes or to maximize the silencing of a singe gene**. a) p6-siRNA can express 6 distinct siRNA to suppress the expression of six different genes (Genes A, B, C, D, E and F). b) p6-siRNA can be used to maximize the efficiency of gene silencing (Gene X) by expressing 6 copies of siRNA targeting the same region of Gene X or by expressing 6 distinct siRNA targeting different regions of Gene X.

Here we report the development and validation of a series of vectors containing multiple siRNA expression cassettes for maximizing the efficiency and versatility of the vector-based gene silencing approach. We consider our current work as an important step forward in the vector-based siRNA technology development. Specifically, the vectors containing multiple siRNA expression cassettes have two important applications: First these vectors can produce different siRNA to knock down the expression of different genes; secondly, they can be used to maximize the silencing of a singe gene. In Fig. 4a, we use p6-siRNA as an example to explain the two major applications. 6 different DNA inserts encoding effective siRNA targeting 6 different genes (Genes A, B, C, D, E and F) may be cloned into p6-siRNA. Once these vectors are delivered into mammalian cells by transient transfection or by viral technology, one single vector would produce 6 different siRNA to induce degradation of 6 different transcripts (Genes A, B, C, D, E and F). This feature is very attractive because many studies such as cell signaling pathway analysis necessitate the silencing of multiple genes.

Moreover, as shown in Fig. 4b, p6-siRNA may be used to maximize the down-regulation of a single gene expression. This can be achieved in two ways. First, DNA inserts containing the same siRNA are cloned into all six siRNA expression cassettes in p6-siRNA vector (left panel, Fig. 4b). Consequently, one such vector would produce six times more siRNA than a vector containing only one siRNA expression cassette, resulting in a dramatic increase in efficiency of gene silencing. Alternatively, 6 different siRNA inserts are cloned into p6-siRNA to produce 6 distinct siRNA to target different regions of the same gene to maximize the down-regulation of its expression (Gene X) (right panel, Fig. 4b).

As shown in Fig. 1b, we developed six vectors with different number of siRNA expression cassettes. We would like to point out that the use of an appropriate vector is very critical for achieving the maximal efficiency of gene silencing. For instance, if one plans to use 4 different siRNA sequences, it is critical to use p4-siRNA rather than p5-siRNA or p6-siRNA despite that both p5-siRNA and p6-siRNA can accommodate 4 different siRNA inserts. If p6-siRNA is used, 4 siRNA cassettes contain DNA inserts and other two remain empty. Once the p6-siRNA is transfected into cells, U6 promoters in all 6 siRNA expression cassettes will recruit Pol III no matter whether they contain an DNA insert or not. As such, the two empty siRNA cassettes would negatively affect the efficiency of the 4 insert-containing cassettes since the two U6 promoters in the empty cassettes would compete with the four U6 promoters in the insert-containing cassettes for Pol III. Thus, in this case, in order to achieve the maximal efficiency of gene silencing, p4-siRNA vector should be used.

The vectors shown in Fig. 1b represent only prototypes of this series of siRNA expression vectors. Using a similar cloning strategy, we may develop additional vectors to improve the power and efficiency of the siRNA vectors containing multiple siRNA expression cassettes. First, since the vectors shown in Fig. 1b utilizes mouse U6 promoter to produce siRNA, these vectors may not be as efficient in human cells as in mouse cells. Thus, we may develop similar vectors for silencing multiple genes in human cells by using human U6 promoter. Secondly, we may also develop vectors in which both mouse U6 promoter and mouse H1 promoter are used to produce siRNA in the same vector. Inclusion of both types of Pol III promoters may increase the efficiency of these vectors. For example, when p6-siRNA shown in Fig. 1b is transfected into cells, 6 U6 promoters will have to compete for the transcription factors available in the cells. In contrast, if p6-siRNA vector contains 3 U6 promoters and 3 H1 promoters, only 3 U6 promoters will have to share the same amount of the transcription factors for the U6 promoter. The other 3 H1 promoters in the vector will recruit the transcription factors for the H1 promoters. As a result, each U6 promoter should have access to more transcription factors (twice more) and this may lead to production of more siRNA.

## Conclusion

Using an efficient cloning strategy, we have constructed siRNA expression vectors containing multiple (up to 6) siRNA expression cassettes. These vectors can be used to simultaneously knock down the expression of different genes or to maximize the gene silencing of a single gene. Thus, these vectors are more powerful and versatile than the siRNA expression vectors currently available. Moreover, using the same cloning strategy, we are capable of constructing additional vectors containing multiple siRNA expression cassettes which may further improve the efficiency of gene silencing in cells of interest.

## Methods

### Construction of siRNA expression vectors

The standard molecular cloning methods were used to construct the vectors containing multiple siRNA expression cassettes with the oligonucleotides shown in Fig. 1a and additional files (see Additional file 1). A mouse U6 promoter (mU6) (from -315 to +1 relative to the transcription start site) was amplified by PCR using mouse genomic DNA using the following pair of primers: the forward primer 5'-taaactagtgacgccgccatctctaggcccgcg-3' and the reverse primer 5'-tatctcgagtctccaagggatatttatagtctccaaaac-3. The forward primer contains a Spe I site at its 5' end (underlined) while the reverse primer has a Xho I site at its 5' end (underlined). The PCR product was cloned into SK+ between Spe I and Xho I to give rise to SK-mU6. The PCR product was confirmed by sequencing.

### Culturing and infection of BMMs

Bone marrow cells were isolated from long bones of 4–8 week old mice (Harlan Industries, Indianapolis, IN) as described [13]. BMMs were prepared by culturing isolated bone marrow cells in α-minimal essential medium containing 10% heat-inactivated FBS in the presence of 0.1 volume of culture supernatant of monocyte/macrophage colony stimulating factor (M-CSF)-producing cells for 2 days as previously described [14].

Retrovirus was prepared using Plat-E packaging cells [15]. Plat-E cells were cultured in Dulbecco's modified eagle medium with 10% heat-inactivated FBS as described previously [15] and transiently transfected with the viral vectors containing multiple siRNA expression cassettes using Lipofectamine Plus (Invitrogen, Carlsbad, CA) as described previously [11]. Virus supernatant was collected at 48, 72 and 96 hours after transfection. BMMs were then infected with virus for 24 hours in the presence of 0.1 volume of culture supernatant of M-CSF-producing cells and 8 μg/ml polybrene. Cells were further cultured in the presence of M-CSF and 2 μg/ml puromycin for selection and expansion of transduced cells. Selected cells were subsequently used for Western analysis.

### Western analysis

BMMs infected with retrovirus or control BMMs (uninfected) were cultured in α-MEM in the presence of M-CSF (22 ng/ml). Cells were washed twice with ice-cold phosphate-buffered saline (PBS) and then lysed in buffer containing 20 mM Tris, pH 7.5, 150 mM NaCl, 1 mM EDTA, 1 mM EGTA, 1% Triton X-100, 2.5 mM sodium pyrophosphate, 1 mM β-glycerophosphate, 1 mM Na_3_VO_4_, 1 mM NaF, and 1× protease inhibitor cocktail 1 (Sigma, P-2850) and 1× protease inhibitor cocktail 2 (Sigma, P-5726). Lysates were then subjected to Western analysis as described in our previous study [11]. Antibodies against TRAF1 (sc-874), TRAF2 (sc-876) and TRAF6 (sc-8409) were purchased from Santa Cruz, Inc (Santa Cruz, CA). Membranes were washed extensively and enhanced chemiluminescence detection assay was performed using SuperSignal West Dura kit from Pierce (Rockford, IL). Quantification of the blots was performed using FlourChem v2.0 from Alpha Innotech Corporation (San Leandro, CA).

## Abbreviations

RNAi, RNA interference; dsRNA, double-stranded RNA; siRNA, small interfering RNA; Pol III, RNA polymerase III; mRNA, messenger RNA; SK+, pBluescript II SK+; MCS, multiple cloning sites; mU6, mouse U6 promoter; BMMs, bone marrow macrophages; M-CSF, monocyte/macrophage colony stimulating factor; PBS, phosphate-buffered saline.

## Authors' contributions

All the listed authors contributed substantially to the study. As a senior author and the PI in the study, XF originated the idea and supervised all the studies. SW developed the vectors. WL identified siRNA sequences that are capable of knocking down TRAF1, TRAF2 or TRAF6 and assisted SW in validation of the vectors. ZS amplified mouse U6 promoter and cloned it into SK and assisted SW in cloning of the promoter into these vectors. ZS and JJ performed assays using the scrambled siRNA controls.

## Supplementary Material

Additional File 1 Construction of p3-siRNA, p4-siRNA, p5-siRNA and p6-siRNA. The data provided describe the detailed procedures for constructing p3-siRNA, p4-siRNA, p5-siRNA and p6-siRNA. Click here for file

Additional File 2 Sequence and structure of Si1, Si2, Si6 and Si6A. The table provided describes the detailed information on sequence and structure of Si1, Si2, Si6 and Si6A. Click here for file
