# Supplementary material for: Development and validation of vectors containing multiple siRNA expression cassettes for maximizing the efficiency of gene silencing
Source: BMC Biotechnol. 2006 Dec 22;6:50. doi: 10.1186/1472-6750-6-50 (PMC1780051; doi:10.1186/1472-6750-6-50)
Supplement: Additional File 1 — Construction of p3-siRNA, p4-siRNA, p5-siRNA and p6-siRNA. The data provided describe the detailed procedures for constructing p3-siRNA, p4-siRNA, p5-siRNA and p6-siRNA. [file 1472-6750-6-50-S1.pdf]

Procedures for Constructing p3-siRNA

p2-siRNA

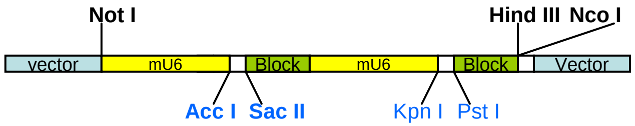

1. Cut **p2-siRNA** with Hind III and Nco I
2. Ligate the following oligos.
3. The resulting plasmid called **Vector 3**

```
       Hind III  Xba I  Sal I   Bgl II Ins BspE I              Block              Mlu I    Nco I
P3-A   5'-AGCTT  TCTAGA GTCGAC  AGATCT TCA TCCGGA ACTATGCTGTGTCTTGACAGCAGACCTCGT ACGCGT C-3'
          3'-A   AGATCT CAGCTG  TCTAGA AGT AGGCCT TGATACGACACAGAACTGTCGTCTGGAGCA TGCGCA GGTAC-5'  P3-B
```

Vector 3

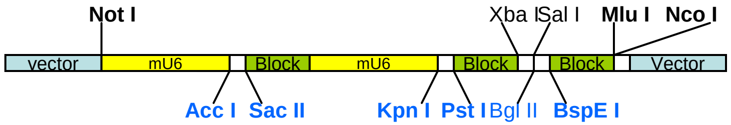

1. Cut Vector 3 with Xba I and Sal I.
2. Cut SK- mU6-promoter with Spe I and Xho I.
3. Ligate Vector 3 and the U6 promoter fragment.
4. The resulting plasmids called **p3-siRNA**

p3-siRNA

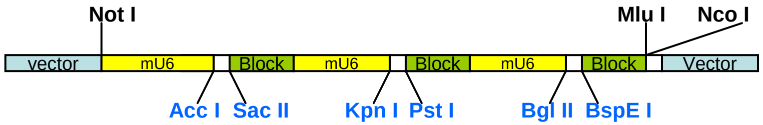

Supplemental Figure: Detailed cloning procedures for constructing p3-siRNA, p4-siRNA, p5-siRNA and p6-siRNA

Procedures for Constructing p4-siRNA

p3-siRNA

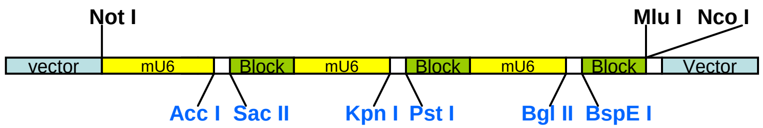

1. Cut **p3-siRNA** with Mlu I and Nco I
2. Ligate the following oligos
3. The resulting plasmid called **Vector 4**

```
         Mlu I  Xba I  Sal I Afl II Ins BsrG I           Block              Xho I   Nco I
P4-A   5'-CGCGT TCTAGA GTCGAC CTTAAG TCA TGTACA TGGACACACAAGTACTGTCGGCAACCACAC CTCGAG C-3'
           3'-A AGATCT CAGCTG GAATTC AGT ACATGT ACCTGTGTGTTCATGACAGCCGTTGGTGTG GAGCTC GGTAC-5'  P4-B
```

Vector 4

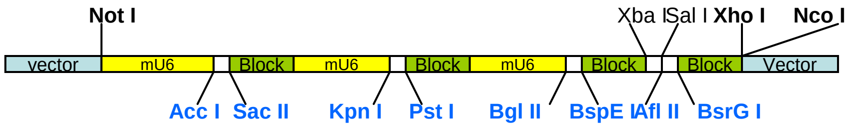

1. Cut Vector 4 with Xba I and Sal I.
2. Cut SK- mU6-promoter with Spe I and Xho I.
3. Ligate Vector and the U6 promoter fragment.
4. The resulting plasmids called **p4-siRNA**

p4-siRNA

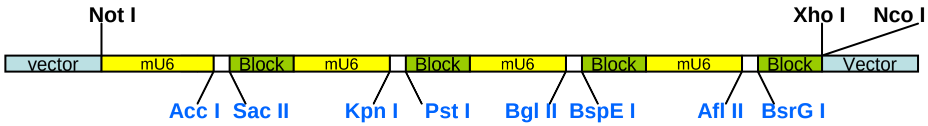

Supplemental Figure: Detailed cloning procedures for constructing p3-siRNA, p4-siRNA, p5-siRNA and p6-siRNA

Procedures for Constructing p5-siRNA

p4-siRNA

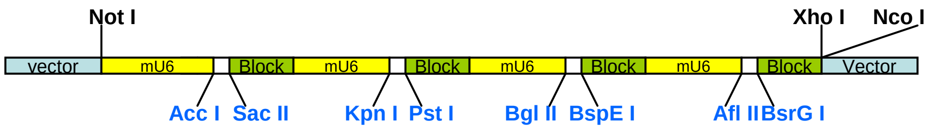

1. Cut **p4-siRNA** with Xho I and Nco I
2. Ligate the following oligos
3. The resulting plasmid called **Vector 5**

```
           Xho I   Xba I  Sal I   Age I  Ins  Sph I              Block                Bam HI   Nco I
P5-A  5'-TCGAG  TCTAGA GTCGAC  ACCGGT TCA GCATGC TGAGAACTGCTAGTCTCGTGACAGCGACTT GGATCC C-3'
          3'-C  AGATCT CAGCTG  TGGCCA AGT CGTACG ACTCTTGACGATCAGAGCACTGTCGCTGAA CCTAGG GGTAC-5'  P5-B
```

Vector 5

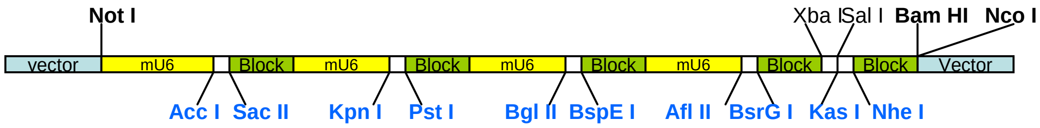

1. Cut Vector 5 with Xba I and Sal I.
2. Cut SK- mU6-promoter with Spe I and Xho I.
3. Ligate Vector 5 and the U6 promoter fragment.
4. The resulting plasmids called **p5-siRNA**

p5-siRNA

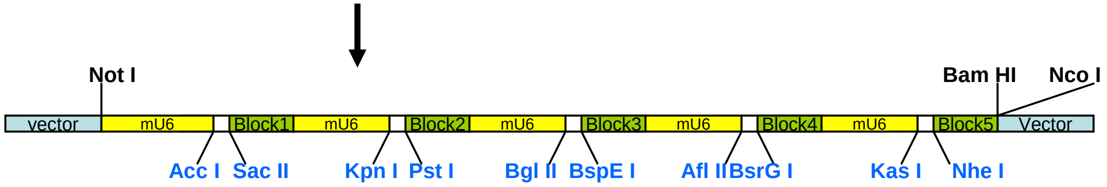

Supplemental Figure: Detailed cloning procedures for constructing p3-siRNA, p4-siRNA, p5-siRNA and p6-siRNA

Procedures for Constructing p6-siRNA

p5-siRNA

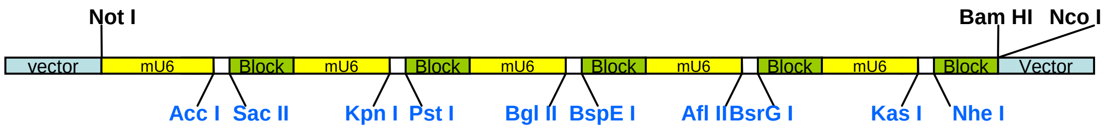

1. Cut **p5-siRNA** with Bam HI and Nco I
2. Ligate the following oligos
3. The resulting plasmid called **Vector 6**

```
        Bam HI  Xba I  Sal I   Aat II Ins  Mfe I          Block                       Nco I
P6-A   5'-GATCC TCTAGA GTCGAC  GACGTC TCA CAATTG CGAACGTTCTGCGACAACTGAGCAGTGAGA C-3'
           3'-G AGATCT CAGCTG  CTGCAG AGT GTTAAC GCTTGCAAGACGCTGTTGACTCGTCACTCT GGTAC-5'   P6-B
```

Vector 6

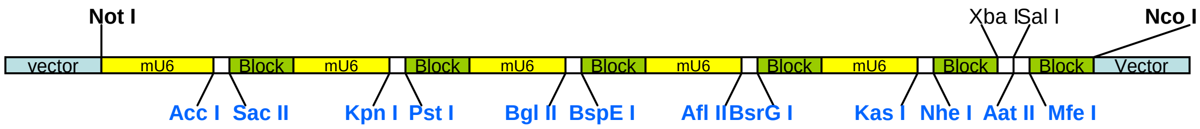

1. Cut Vector 6 with Xba I and Sal I.
2. Cut SK-mU6-promoter with Spe I and Xho I.
3. Ligate Vector 6 and the U6 promoter fragment.
4. The resulting plasmids called **p6-siRNA**

p6-siRNA

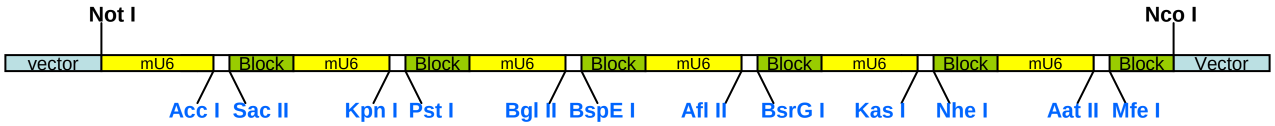

Supplemental Figure: Detailed cloning procedures for constructing p3-siRNA, p4-siRNA, p5-siRNA and p6-siRNA
