# Supplementary material for: Development and validation of vectors containing multiple siRNA expression cassettes for maximizing the efficiency of gene silencing
Source: BMC Biotechnol. 2006 Dec 22;6:50. doi: 10.1186/1472-6750-6-50 (PMC1780051; doi:10.1186/1472-6750-6-50)
Supplement: Additional File 2 — Sequence and structure of Si1, Si2, Si6 and Si6A. The table provided describes the detailed information on sequence and structure of Si1, Si2, Si6 and Si6A. [file 1472-6750-6-50-S2.pdf]

Table 1. Sequence and structure of Si1, Si2, Si6 and Si6A

siRNA sequence	19 nt coding sequence	Spacer	19 nt complementary sequence	Stop signal
**Si1**	5′-AGATGACACAATGTTCCTC<br>3′-TCTACTGTGTTACAAGGAG	TTCAAGAGA<br>AAGTTCTAT	GAGGAACATTGTGTCATCT<br>CTCCTTGTAACACAGTAGA	TTTTTT-3′<br>AAAAAA-5′
**Si2**	5′-GGTAACATTGATGTTGCTG<br>3′-CCATTGTAACTACAACGAC	TTCAAGAGA<br>AAGTTCTCT	CAGCAACATCAATGTTACC<br>GTCGTTGTAGTTACAATGG	TTTTTT-3′<br>AAAAAA-5′
**Si6**	5′-GGATGATACATTACTAGTG<br>3′-CCTACTATGTAATGATCAC	TTCAAGAGA<br>AAGTTCTCT	CACTAGTAATGTATCATCC<br>GTGATCATTACATAGTAGG	TTTTTT-3′<br>AAAAAA-5′
**Si6A**	5′-TACTGTGGTACAATCCTCA<br>3′-ATGACACCATGTTAGGAGT	TTCAAGAGA<br>AAGTTCTCT	TGAGGATTGTACCACAGTA<br>ACTCCTAACATGGTGTCAT	TTTTTT-3′<br>AAAAAA-5′
